# A retrospective review in the management of T3 laryngeal squamous cell carcinoma: an expanding indication for transoral laser microsurgery

**DOI:** 10.1186/s40463-016-0147-1

**Published:** 2016-05-27

**Authors:** A. Butler, M. H. Rigby, J. Scott, J. Trites, R. Hart, S. M. Taylor

**Affiliations:** Dalhousie University, Halifax, Canada

**Keywords:** Laryngeal, Carcinoma, Transoral, Laser, Glottic

## Abstract

**Background:**

The purpose of this study was to evaluate the functional and oncological outcomes of patients treated for T3 laryngeal squamous cell carcinoma. Specifically comparing transoral laser microsurgery and radiotherapy/chemoradiotherapy treatment modalities.

**Method:**

A retrospective review of patients treated for T3 laryngeal SCC between 2002 and 2010 was undertaken.

**Results:**

Forty-nine patients were included. 15 cases were glottic, (9 treated with TLM, 6 with RT/CRT), 33 supraglottic (6 treated with TLM, 27 with RT/CRT) and 1 subglottic subsite (treated with RT/CRT). There was no statistical difference between treatment groups for 24 month locoregional control (72.3 %), overall survival (glottis 86.7 %, supraglottic 70.4 %) and disease specific survival (glottic 93.3 % and supraglottic 74.1 %). Overall laryngeal preservation (84.9 %) was also similar in both groups.

**Conclusion:**

Our institution is expanding the application of TLM to selected patients with T3 laryngeal carcinoma. Oncological outcomes have not been jeopardized by this approach and the treatment is well tolerated by patients with few complications.

## Background

Laryngeal cancer is the second most frequent head and neck cancer. The overwhelming majority of these cancers are squamous cell carcinomas. These cancers are associated with both tobacco and alcohol use and, while this is changing, have historically occurred predominantly in males. For the 2010 Canadian population, laryngeal cancer had an estimated incidence rate of 2.0/100 000 person*years for males and 0.9/100 000 person*years in females [1]. U.S data has demonstrated that over the past 30 years, laryngeal cancer has the distinction of being one of the few cancers of the head and neck that has shown a decrease in survival [2, 3].

Transoral laser microsurgery (TLM), originally introduced in 1972 by Jako and Strong [1], has consistently been shown to have good oncological and functional outcomes in patients with early glottic cancer. Steiner et al,. reported oncological and functional results in the treatment of T3 laryngeal tumours equivalent to radiotherapy (RT)/chemoradiotherapy(CRT) and open laryngeal surgery, which expanded the indications for TLM in laryngeal cancer [2]. Despite this, the management of T3 glottic carcinoma remains controversial. Most commonly, such tumours are treated with radical radiotherapy with adjuvant chemotherapy, open partial resection, or total laryngectomy.

Although recent studies support a possible role for a primary surgical approach, few centers such as the QEII Health Sciences Centre in Halifax, Nova Scotia have incorporated the use of TLM in the management of T3 glottic tumours [2]. The purpose of this paper is to review all T3 laryngeal squamous cell carcinomas (SCC) treated at the QEII Health Sciences Centre in Halifax, Nova Scotia, and to compare those patients treated with TLM to those managed with radiotherapy/chemoradiotherapy.

## Method

Data was obtained from the senior authors prospective database of malignancies treated with TLM at the QEII Health Sciences Centre in Halifax, Nova Scotia. Our institutional research ethics board approved the collection of information within the database. The details of this database have been previously described [4]. It has been prospectively maintained since 2005, and data prior to 2005 included retrospectively.

Inclusion criteria included all patients who underwent TLM or RT/CRT with curative intent for previously untreated T3 laryngeal SCC between January 2002 and December 2010. Exclusion criteria included all other sub-sites and stages, and recurrent T3 glottic SCC.

A retrospective cohort analysis was performed including a descriptive analysis of demographics and oncological outcomes. Demographics recorded included age, gender and smoking status. ANOVA test for difference in means and the Fisher Exact test were performed to identify any differences between the two therapeutic groups. Tumours were staged according to TNM American Joint Committee on Cancer (AJCC) guidelines [5]. Tumours excised with TLM were staged pathologically. Treatment modality and date of treatment commencement were recorded. Any treatment morbidity or complications were identified. Survival analysis using Kaplan Meier curve was performed, assessing local, regional and distant metastatic control, disease free survival, disease specific survival, overall survival and laryngeal preservation. Two year outcomes were recorded as 5 year data was not yet available.

All patients were treated with either transoral laser microsurgery +/- adjunctive radiation therapy (TLM), chemoradiation (CRT) or radiation therapy (RT) alone. Allocation to treatment modality was not randomized and was based on patient preference and recommendation of the site Head and Neck Oncology Tumour Board. Not all patients were considered eligible for all treatment modalities. Patients undergoing radiation alone either refused chemotherapy, or had a medical contraindication. Transoral laser surgery was performed using a tumour splitting technique with a CO_2_ laser. Frozen sections were performed intraoperatively, and positive margins post-operatively were re-resected. Radiation therapy was performed using intensity modulated radiation therapy with 70 Gy with standard fractionation. Concurrent chemotherapy consisted of Cisplatinum with or without 5-fluorouracil given on weeks 1, 4 and 7 concurrently with radiation.

Functional outcomes of voice and swallowing were documented as assessed at the last follow up review, using Voice Handicap Index (VHI) 10 and Functional Outcome Swallowing Study (FOSS) respectively. No scores were available prior to treatment commencement. Results were analysed using the Wilcoxon significance rank test.

## Results

Forty-nine patients met the inclusion criteria of the study. Fifteen (30.6 %) were treated with TLM and the remaining 34 (69.4 %) were treated with RT/CRT. No patients underwent open laryngeal surgery for primary treatment. 15 cases were glottic, (9 treated with TLM, 6 with RT/CRT), 33 supraglottic (6 treated with TLM, 27 with RT/CRT) and 1 subglottic subsite (treated with RT/CRT). Table 1

**Table 1:** Treatment according to subsite

Primary treatment modality	TLM			RT		Total
Adjuvent treatment		RT	CT		CT	
Glottic	9	4	0	6	4	15
Supraglottic	6	3	2	27	20	33
Subglottic				1		1

.

Thirty-eight patients (77.6 %) were male, with no difference in gender distribution between the two treatment groups (p-value 0.5). The mean age was 69.1 years (61.1 years TLM, 66.5 years RT/CRT, age difference between treatment groups *p* value 0.39). Mean follow-up duration for those treated with TLM was 29 months, and 41 months for those treated with RT/CRT. Thirty-three patients were staged as N0, eight as N1 and eight as N2. No patients presented with distant metastasis. The majority of patients with nodal metastasis received RT/CRT (17) as primary treatment rather than TLM (3). Seven (46.7 %) of TLM patients underwent adjuvant RT. None received adjuvant chemotherapy. Twenty-four (77.4 %) patients who underwent RT received concurrent chemotherapy. There were no statistically significant differences in the parameters of age, gender, smoking status, nodal status and date of initial treatment between treatment modalities. Table 2.

**Table 2:** Patient age, gender, nodal staging, smoking status and date of treatment according to treatment

	All	TLM	RT	CRT	*p*
Number	49	15	10	24	-
Age					0.39^a^
Mean	63.1	61.1	66.5	63.0	
(SD)	(9.6)	(11.2)	(7.5)	(9.4)	
Gender					0.50^b^
Female	11	3	1	7	
Male	38	12	9	17	
(% Male)	(78 %)	(80 %)	(90 %)	(71 %)	
Nodal stage					0.36^b^
0–I	41	14	9	18	
II–III	8	1	1	6	
(%II–III)	(16 %)	(7 %)	(10 %)	(25 %)	
Smoking					0.67^b^
No	16	5	5	7	
Yes	10	4	1	5	
Unknown	23	6	4	12	
(% Yes^c^)	(37 %)	(44 %)	(17 %)	(42 %)	
Date of initial treatment					0.30^b^
2002–06	24	5	5	14	
2007–11	25	10	5	10	
(% 2007–11)	(51 %)	(67 %)	(50 %)	(42 %)	

^a^ANOVA F-test for means TLM, RT, CRT

^b^Fisher exact test for table TLM, RT, CRT

^c^% of those with known smoking status

### Locoregional control

**Fig. 1:**
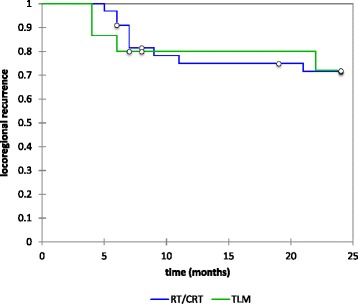
Two-year Kaplan-Meier estimates for overall locoregional control (72.3 %, no difference between either treatment group, *p* value 0.94))

Thirteen patients in the study group developed a locoregional recurrence (26.5 %). The Kaplan-Meier estimates for 24 month locoregional control was 72.3 % in the entire cohort. There was no difference between the treatment groups (72 % TLM, 71.6 % RT/CRT *p* value 0.94) Fig. 1.

In patients with glottic cancer, the Kaplan Meier estimate for locoregional control was 86.7 %. Two patients in the glottic group developed locoregional recurrence (12.5 %). Although they were both treated with TLM, this was not statistically significant compared to those treated with RT/CRT (*p* value 0.98) Both patients were managed with a salvage laryngectomy.

The Kaplan Meier estimate for locoregional control in patients with supraglottic cancer was 64.5 %, with no statistically significant difference between treatment groups (*p* value 0.98). Of the 11 patients (33.3 %) with recurrence, 2 were treated with TLM (33.3 %) and 9 with RT/CRT (33.3 %). Both patients treated with TLM who developed recurrence, were successfully treated with subsequent radiotherapy. Those patients with recurrence treated initially with RT/CRT, 2 were managed successfully with TLM, 1 was treated palliatively, and the remaining patients underwent salvage total laryngectomy.

### Survival

**Fig. 2:**
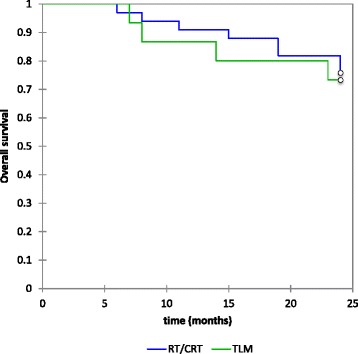
Two-year Kaplan-Meier estimates for overall survival (TLM 73.3 %, RT/CRT 75.8 %, *p* value 0.8)

Of the 49 patients, 12 (24.5 %) died within 24 months. Of these, 9(18.3 %) deaths were disease-related. Kaplan-Meier estimates showed 2 year overall survival was 75 % and again similar between the two treatment groups with 73.3 %% in the TLM group and 75.8 % RT/CRT group (*p* value 0.8) Fig. 2. Glottic overall survival was 86.7 % while supraglottic overall survival was 70.4 %. Neither group showed a statistical difference between treatment regimens.

**Fig. 3:**
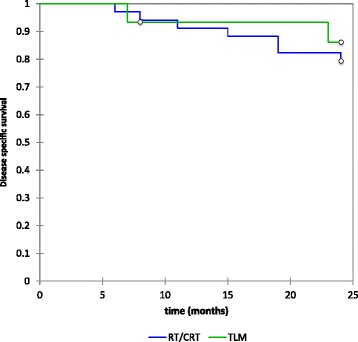
Two-year Kaplan-Meier estimates for overall disease specific survival (81.4 %, no difference between either treatment group, *p* value 0.60)

Overall disease specific survival was 81.4 % and was also similar for those treated with TLM (86.2 %), and those treated with RT/CRT (79.4), (*p* value 0.60) Fig. 3. Glottic DSS was 93.3 % and supraglottic DSS was 74.1 %. Again neither group showed a statistically significant difference between treatment regimens (*p* value 0.41 and 0.75 respectively).

### Laryngeal preservation

**Fig. 4:**
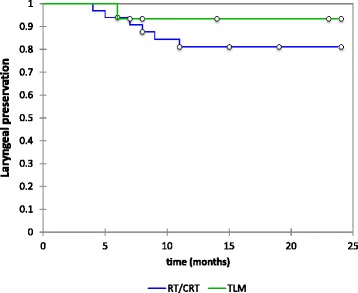
Two-year Kaplan-Meier estimates for overall laryngeal preservation (84.9 %, no difference between either treatment group, *p* value 0.35)

Overall laryngeal preservation rates (84.9 %) trended towards being greater in the TLM group (93 vs 81 %), but this was not statistically significant. (*p* value 0.35) Fig. 4. Glottic laryngeal preservation was 86.7 % and supraglottic laryngeal preservation 83.9 %. Although there was not a statistical difference between TLM and RT/CRT in either subsite, it is worth noting that no patients treated with TLM in the supraglottic group required a salvage laryngectomy.

### Post-operative complications and functional results

Voice Handicap Index (VHI) and Functional Outcome Swallow Scale (FOSS) scores were available in only a limited number of patients. For glottic cases, the median function outcomes at last post-treatment visit trended towards worse voice scores (VHI 10) for those treated with TLM (15 TLM, 11 RT/CRT) but this was not statistically significant. However, for supraglottic cases, VHI trended towards better in those treated with TLM (9 TLM, 11.5 RT/CRT). Median FOSS scores were identical.

In the TLM glottic group one patient developed a small post-operative hemorrhage, which did not require any intervention. No patients required temporary or permanent tracheostomy, and none required nasogastric feeding. One patient in the RT/CRT group required nasogastric nutrition and three patients were unable to tolerate chemotherapy and the treatment discontinued.

## Discussion

Following the Department of Veterans Affairs Laryngeal Cancer Group trial the management of laryngeal cancer shifted towards chemoradiation [6]. During the 1990’s it was noted by Hoffman et al. that with the increased use of CRT, the 5-year survival for T3N0M0 laryngeal cancer had declined [7]. More recently, there continues to be much debate regarding best treatment practices for advanced laryngeal tumours, in particular a move back towards primary surgical treatment.

Total laryngectomy (TL) is frequently used to treat T3 glottic carcinoma with or without (chemo) radiotherapy. Although good oncological results are achieved with TL, the operation has obvious functional sequelae. Published 5-year locoregional control and overall survival range from 69 to 87 % and 53 to 56 % respectively [8–10]. Open partial laryngectomy also has good locoregional control and overall survival with 5-year rates between 73 and 83 % and 71 to75% respectively [11, 12]. In this study, our locoregional control and overall survival for T3 glottic carcinomas treated with TLM are similar to that of open laryngeal procedures, with locoregional control of 87 % and 2 year overall survival of 68 %.

Radiotherapy alone in the absence of chemotherapy has been shown to have poorer outcomes. Chemotherapy, however, is contraindicated in patients with poorer general health. In patients who therefore have a favourable tumour location, yet in whom chemotherapy is a contraindication, TLM is without question a reasonable surgical option.

As this was not a randomized study, those patients selected for TLM may have had favourable anatomy and tumour location. Because of this fact, a direct comparison of the two groups should be interpreted cautiously. However, management with TLM did not appear to jeopardize oncological outcomes in those selected for this minimally invasive approach.

The low numbers within groups make the study underpowered to detect clinically significant differences between treatments. However, adequate sample size for this study to power the log rank test this to be able to detect the difference between a 5-year survival of 0.8 to 0.7 are would require over 1000 cases, and would be difficult to obtain this sample size outside of a national database.

The cases in this trial were not selected for treatment by patient and tumour characteristics and were not randomized. There are two potential differences in the characteristics of persons between treatment modalities that, while not statistically significant, are concerning with respect to the comparability of treatment groups. The first is the increased mean age of those undergoing radiation therapy alone. This would be expected as age greater then 70–75 is a relative contraindication to CRT. This is the cutoff that has been excluded from most CRT trials, and there is a higher rate of significant morbidity associated with CRT in those over 70–75. The second is the fact that 75 % of all N2 and N3 disease was treated by CRT. N2 or N3 disease upstages T3 laryngeal cancer to stage IVa and IVb respectively. This relative overrepresentation of more advanced disease in the CRT group may underestimate the efficacy of this modality when compared to TLM and RT groups.

Although this study showed reasonably low complication rates in those receiving chemoradiotherapy, chemotherapy in addition to the radiotherapy is known to have associated significant toxicity with mucositis, leukopenia and a 2 % increase in early deaths secondary to acute toxicity [13–15]. As complications were not prospectively recorded, mucositis and leukopenia were not accurately documented in this study and it is likely that compared to surgical complications, they were underreported.

When locoregional recurrence does occur in patients treated with TLM, options for management include further TLM, RT/CRT or open partial or total laryngectomy. For patients treated primarily with RT/CRT, although salvage surgery is possible the rate of complications in particular pharyngocutaneous fistula, the need for microvascular or regional flaps, complexity of surgery and the overall cost of prolonged hospital stay significantly increase [16, 17]. Complications of salvage treatment were not specifically reviewed in this study. Anecdotal evidence suggests complications from salvage surgery are likely to be less if radiotherapy can be avoided.

Over the study period, the senior authors selection of patients for TLM has changed. As surgical experience in TLM increased for early glottic carcinoma and expertise has developed, so too was there an increase in the T3 cases treated with TLM. Bernal-Sorekelsen et al. reported that for locally advanced tumours, a significant difference in the experience of the surgeon altered the complication rate, in particular bleeding and aspiration pneumonia [18]. They recommended for TLM “beginners” even when experienced in external approaches, to accumulate a significant experience with early staged tumors before attempting to treat advanced cases in this manner.

It has been the experience of the senior author, and also discussed in the literature, that T3 glottic tumours with vocal cord fixation are more likely to develop local recurrence [19]. Cord fixation may be due to a number of factors, including cricoarytenoid joint involvement, muscle infiltration or paraglottic space involvement. Whilst true cord fixation is both a poorer prognostic factor for TLM and more likely to result in post-operative aspiration, paraglottic involvement does not carry such a poor prognosis. Therefore careful pre-operative image review and clinical assessment of the patient is imperative. As noted by Vilaseca et all, pre-epiglottic space involvement is associated with a better prognosis for TLM, as the pre-epiglottic space can be easily exposed and wider margins achieved with good functional outcomes. It is our opinion that the same can be said for isolated paraglottic space invasion in T3 glottic cancer.

## Conclusion

The use of transoral laser microsurgery for advanced laryngeal cancer is controversial. With over a decade of increasing experience with TLM for early glottic cancer, our institution is expanding the application of TLM to selected T3 glottic carcinoma. Oncological outcomes are not jeopardized by this approach and the treatment is well tolerated by patients with few complications. The data reported herein supports the ongoing use of TLM for selected T3 glottic cancers at our centre.

## Abbreviations

CRT, chemoradiotherapy; FOSS, functional outcome swallow study; RT, radiotherapy; SCC, squamous cell carcinoma; TLM, transoral laser microsurgery; U.S, United States; VHI, voice handicap index
